# Carbonate Apatite Nanoparticles Act as Potent Vaccine Adjuvant Delivery Vehicles by Enhancing Cytokine Production Induced by Encapsulated Cytosine-Phosphate-Guanine Oligodeoxynucleotides

**DOI:** 10.3389/fimmu.2018.00783

**Published:** 2018-04-18

**Authors:** Hideki Takahashi, Kazuki Misato, Taiki Aoshi, Yasuyuki Yamamoto, Yui Kubota, Xin Wu, Etsushi Kuroda, Ken J. Ishii, Hirofumi Yamamoto, Yasuo Yoshioka

**Affiliations:** ^1^Vaccine Creation Project, BIKEN Innovative Vaccine Research Alliance Laboratories, Research Institute for Microbial Diseases, Osaka University, Suita, Japan; ^2^Vaccine Dynamics Project, BIKEN Innovative Vaccine Research Alliance Laboratories, Research Institute for Microbial Diseases, Osaka University, Suita, Japan; ^3^Vaccine Dynamics Project, BIKEN Center for Innovative Vaccine Research and Development, The Research Foundation for Microbial Diseases of Osaka University, Suita, Japan; ^4^Vaccine Creation Project, BIKEN Center for Innovative Vaccine Research and Development, The Research Foundation for Microbial Diseases of Osaka University, Suita, Japan; ^5^Division of Health Sciences, Department of Molecular Pathology, Graduate School of Medicine, Osaka University, Suita, Japan; ^6^Laboratory of Adjuvant Innovation, Center for Vaccine and Adjuvant Research, National Institutes of Biomedical Innovation, Health and Nutrition, Ibaraki, Japan; ^7^Laboratory of Vaccine Science, WPI Immunology Frontier Research Center, Osaka University, Suita, Japan; ^8^Laboratory of Nano-Design for Innovative Drug Development, Graduate School of Pharmaceutical Sciences, Osaka University, Suita, Japan; ^9^Global Center for Medical Engineering and Informatics, Osaka University, Suita, Japan

**Keywords:** adjuvant, cytosine-phosphate-guanine oligodeoxynucleotide, influenza virus, interferon-α, nanoparticle, vaccine

## Abstract

Vaccine adjuvants that can induce not only antigen-specific antibody responses but also Th1-type immune responses and CD8^+^ cytotoxic T lymphocyte responses are needed for the development of vaccines against infectious diseases and cancer. Of many available adjuvants, oligodeoxynucleotides (ODNs) with unmethylated cytosine-phosphate-guanine (CpG) motifs are the most promising for inducing the necessary immune responses, and these adjuvants are currently under clinical trials in humans. However, the development of novel delivery vehicles that enhance the adjuvant effects of CpG ODNs, subsequently increasing the production of cytokines such as type-I interferons (IFNs), is highly desirable. In this study, we demonstrate the potential of pH-responsive biodegradable carbonate apatite (CA) nanoparticles as CpG ODN delivery vehicles that can enhance the production of type-I IFNs (such as IFN-α) relative to that induced by CpG ODNs and can augment the adjuvant effects of CpG ODNs *in vivo*. In contrast to CpG ODNs, CA nanoparticles containing CpG ODNs (designated CA-CpG) induced significant IFN-α production by mouse dendritic cells and human peripheral blood mononuclear cells *in vitro*; and production of interleukin-12, and IFN-γ was higher in CA-CpG-treated groups than in CpG ODNs groups. In addition, treatment with CA-CpG resulted in higher cytokine production in draining lymph nodes than did treatment with CpG ODNs *in vivo*. Furthermore, vaccination with CA-CpG plus an antigen, such as ovalbumin or influenza virus hemagglutinin, resulted in higher antigen-specific antibody responses and CD8^+^ cytotoxic T lymphocyte responses *in vivo*, in an interleukin-12- and type-I IFN-dependent manner, than did vaccination with the antigen plus CpG ODNs; in addition, the efficacy of the vaccine against influenza virus was higher with CA-CpG as the adjuvant than with CpG ODNs as the adjuvant. These data show the potential of CA nanoparticles to serve as CpG ODN delivery vehicles that increase the production of cytokines, especially IFN-α, induced by CpG ODNs and thus augment the efficacy of CpG ODNs as adjuvants. We expect that the strategy reported herein will facilitate the design and development of novel adjuvant delivery vehicles for vaccines.

## Introduction

As the recent Ebola virus outbreak and past worldwide influenza pandemics have demonstrated, infectious diseases are a serious global health problem (1). Vaccines are the most efficient way to prevent infectious diseases, and as a result of immunological innovations such as the development of checkpoint inhibitors, vaccines are also expected to be effective against cancer in the coming years (2).

There are various types of vaccines, including attenuated live vaccines, inactivated whole vaccines, and protein- or peptide-based subunit vaccines (3–5), and they all have various advantages and disadvantages. For example, although attenuated live vaccines can generate strong adaptive immunity, the pathogens in these vaccines have the potential to cause infectious disease in rare cases, especially in individuals with compromised immune systems (3–5). In contrast, protein- or peptide-based subunit vaccines are much safer than attenuated live vaccines and inactivated whole vaccines; however, when used alone, subunit vaccines evoke only weak adaptive immunity. The efficacy of subunit vaccines can be improved by using adjuvants (6). Aluminum salts (alum) are the most widely used adjuvants and are present in many important vaccines, including the diphtheria–tetanus–pertussis-inactivated poliomyelitis vaccine, the pneumococcal conjugate vaccine, and hepatitis B vaccine in humans (7). Unfortunately, although alum induces strong Th2-type immune responses, it cannot induce Th1-type immune responses or CD8^+^ cytotoxic T lymphocyte (CTL) responses, which are necessary for vaccines against infectious diseases such as influenza virus, hepatitis C, and malaria as well as for cancer vaccines designed to eliminate infected or malignant cells (8). Therefore, the development of adjuvants that can induce Th1-type immune responses and CD8^+^ CTL responses is necessary.

Oligodeoxynucleotides (ODNs) with unmethylated cytosine-phosphate-guanine (CpG) motifs, which act as Toll-like receptor 9 (TLR9) agonists, are among the most effective adjuvants for inducing Th1-type immune responses and CD8^+^ CTL responses (9). CpG ODNs, which are short single-stranded synthetic DNA fragments containing immunostimulatory CpG motifs, bind to TLR9 at endosomes after being taken up by B cells and dendritic cells (DCs). This binding initiates an innate immune response characterized by the production of cytokines that can promote Th1-type immune responses. Therefore, CpG ODNs are expected to be useful as vaccine adjuvants and immunotherapeutic agents against infectious diseases and cancer (9). In fact, in Phase II clinical trials, a hepatitis B vaccine with CpG ODNs as an adjuvant has demonstrated superiority to a currently licensed hepatitis B virus vaccine with alum as an adjuvant (10, 11). There are at least four types of CpG ODNs, each of which has a different structure and different physical and immunostimulatory properties (9). Of the various types, D-type CpG ODNs (also known as A-type CpG ODNs) and K-type CpG ODNs (also known as B-type CpG ODNs) are the major types for experimental and clinical uses. D-type CpG ODNs can induce plasmacytoid DCs to produce massive amounts of type-I interferons (IFNs) such as IFN-α. IFN-α is important for the activation and cytotoxicity of natural killer T cells (12), for CD8^+^ T-cell activation (13), and for the maturation of DCs, thus indicating the importance of adjuvants that can induce IFN-α production. D-type CpG ODNs self-assemble into multimers because of their palindromic and poly-G sequences, and these multimers are important for IFN-α induction (14). However, uncontrollable aggregation hinders the clinical utility of D-type CpG ODNs. In contrast, K-type CpG ODNs can activate the production of interleukin (IL)-6 by B cells and DCs but only weakly induce IFN-α production by plasmacytoid DCs. These differences in cytokine production between D-type and K-type CpG ODNs are thought to be the reason for their different adjuvant effects in the treatment of some infectious diseases. For example, K-type CpG ODNs induce better antibody production and cytokine responses than D-type CpG ODNs when used in a malaria vaccine in cynomolgus monkeys (15), whereas D-type CpG ODNs elicit better immune responses than K-type CpG ODNs in a leishmania vaccine in rhesus monkeys (16). In addition, when used as a monotherapeutic for leishmaniasis, D-type CpG ODNs have better therapeutic effects than K-type CpG ODNs (17). Therefore, the design of CpG ODN-based adjuvants with the functions of both D-type and K-type CpG ODNs would be highly desirable.

To design such multifunctional CpG ODNs, researchers have tried to use multimerization to give K-type CpG ODNs the ability to induce IFN-α production. There are two main methods for multimerizing K-type CpG ODNs: the use of biological molecules and the use of synthetic particles. With regard to the former, several types of peptides (e.g., antimicrobial peptide LL37 and Tat peptide) and β-glucan have been used to enhance the ability of K-type CpG ODNs to induce IFN-α production and to increase their adjuvant activity both *in vitro* and *in vivo* (18–20). In contrast, only limited data are available on the use of synthetic particles to enhance the adjuvant activity of K-type CpG ODNs *in vivo* by giving them the ability to induce IFN-α production, although the encapsulation of K-type CpG ODNs in, or their conjugation with, various types of particles has been shown to enhance IFN-α production *in vitro* (14, 21, 22). Therefore, the development of novel particles that contain encapsulated K-type CpG ODNs and can induce IFN-α production would be useful and would facilitate investigations of the relationship between IFN-α production *in vitro* and vaccine effects *in vivo*.

Among the synthetic particles that have been studied to date, pH-sensitive carbonate apatite (CA) particles have been shown to serve as efficient intracellular delivery vehicles for small molecules, as well as for DNA, RNA, and proteins (23–25). For example, the DNA transfection efficiency of CA nanoparticles is 10–100 times that of a general transfection reagent, Lipofectamine, and calcium phosphate precipitation in mammalian cells *in vitro* (23). Furthermore, Yamamoto and colleagues recently developed CA nanoparticles of 10–20 nm diameters by modifying the process by which the nanoparticles were generated (26). Wu et al. reported a simple method for generating CA nanoparticles in which siRNA is encapsulated with high efficiency (26). The resulting CA nanoparticles are highly stable at pH 7.4 but quickly degrade in acidic environments such as endosomal compartments in cells. These siRNA-containing CA nanoparticles show dramatic antitumor effects after intravenous administration and are nontoxic in mice and monkeys (26). These facts suggest that CA nanoparticles have the potential to serve as CpG ODN delivery vehicles owing to their high encapsulation efficiency, low cost, biodegradability, safety, and biocompatibility.

In this study, we show the usefulness of CA nanoparticles as CpG ODN delivery vehicles that give K-type CpG ODNs the ability to induce IFN-α production. We found that CA nanoparticles containing K-type CpG ODNs (designated CA-CpG) showed stronger antigen-specific antibody responses and CD8^+^ CTL responses than those of K-type CpG ODNs, and when mice were vaccinated with the nanoparticles in combination with an antigen, the preventive vaccine effects against influenza virus were better than those of the antigen plus K-type CpG ODNs. This is the first report of the use of pH-responsive biodegradable CA nanoparticles to improve the cytokine production pattern of K-type CpG ODNs and enhance their adjuvant activity.

## Materials and Methods

### Reagents

K-type CpG ODNs (CpG K3: 5′-atcgactctcgagcgttctc-3′) and Alexa 488-labeled K-type CpG ODNs (Alexa 488-labeled CpG K3) were purchased from GeneDesign (Osaka, Japan). EndoGrade endotoxin-free ovalbumin (OVA) (<0.1 EU/mg protein) purchased from Hyglos GmbH (Bayern, Germany) was used for immunization. Grade V OVA purchased from Sigma-Aldrich (St. Louis, MO, USA) was used for enzyme-linked immunosorbent assays (ELISA). Horseradish peroxidase (HRP)-conjugated goat anti-mouse IgG and IgG1 antibodies were purchased from Merck Millipore (Darmstadt, Germany). HRP-conjugated goat anti-mouse IgG2c was purchased from GeneTex, Inc. (Irvine, CA, USA). Alhydrogel adjuvant 2% (alum) was purchased from InvivoGen (San Diego, CA, USA).

### Mice

C57BL/6J mice were purchased from SLC (Kyoto, Japan). *IFN-*α*/*β* receptor 2 (Ifnar2)-Il-12 p40* double-deficient mice have been described previously (27) and were kindly provided by Dr. Ishii (National Institutes of Biomedical Innovation, Health and Nutrition, Ibaraki, Osaka, Japan). Mice were used at 7 to 8 weeks of age and were housed in a room with a 12-h-light/12-h-dark cycle (lights on at 8:00 am, lights off at 8:00 pm) with access to food and water. All animal experiments were performed in accordance with institutional guidelines of Osaka University for the ethical treatment of animals (protocol number H26-11-0).

### Synthesis of CA Nanoparticles and CA-CpG

Carbonate apatite nanoparticles and CA-CpG were prepared as described previously (26). We incubated a 25 mL solution of NaHCO_3_ (44 mM), NaH_2_PO_4_ (0.9 mM), and CaCl_2_ (1.8 mM) (pH 7.5) without or with 1.9, 5.6, 16.7, 50, or 150 µg of K-type CpG ODNs at 37°C for 30 min. The solution was then centrifuged at 12,000 rpm for 3 min, and the pellet was dissolved in saline containing 0.5% mouse serum. The resulting solution of CA nanoparticles or CA-CpG was sonicated in a water bath for 10 min. For determination of the amount of endotoxin, the collected CA-CpG was dissolved in 1 mL of 0.02 M ethylenediaminetetraacetic acid and the amount of endotoxin was determined by means of a Toxicolor LS-50M kit (Seikagaku Corporation, Tokyo, Japan). CA-CpG was found to include <0.2 EU/mL of endotoxin.

### Efficiency of K-Type CpG ODN Encapsulation Into CA Nanoparticles

For determination of the K-type CpG ODN encapsulation efficiency, the collected CA-CpG was dissolved in 1 mL of 0.02 M ethylenediaminetetraacetic acid and the amount of K-type CpG ODNs was determined with a Qubit ssDNA Assay Kit (Thermo Fisher Scientific, Waltham, MA, USA).

### Properties of CA-CpG and CA Nanoparticles

The size distributions of CA nanoparticles and CA-CpG were analyzed with a Zetasizer Nano ZS (Malvern Instruments, Malvern, Worcestershire, UK). Specifically, the mean diameters and particle size distributions of the nanoparticles in mouse serum albumin (Sigma-Aldrich) were measured by means of a dynamic light-scattering method using capillary cells. The size distributions of both types of nanoparticles were also analyzed by atomic force microscopy conducted with a scanning probe microscope (SPM-9500, Shimadzu, Kyoto, Japan) operated in dynamic mode and equipped with a microcantilever (OMCL-AC240TS-R3, Olympus, Tokyo, Japan).

### Preparation and Stimulation of Mouse Bone Marrow-Derived DCs and Human Peripheral Blood Mononuclear Cells

To generate bone marrow-derived DCs, we isolated bone marrow cells from the femurs of C57BL/6J mice and cultured the cells for 7 days with 100 ng/mL human Flt-3L (PeproTech, Rocky Hill, NJ, USA). Cells were seeded at a density of 5 × 10^5^ cells/well in a 96-well flat-bottom culture plate (Nunc, Roskilde, Denmark) and were cultured in complete RPMI medium (RPMI 1640 supplemented with 10 vol% fetal calf serum (FCS), penicillin, and streptomycin). These cells were stimulated with K-type CpG ODNs or CA-CpG for 24 h. Supernatants were subjected to ELISA to determine the levels of IFN-α (PBL Assay Science, Piscataway, NJ, USA), IFN-γ (BioLegend, San Diego, CA, USA), and IL-12 p40 (BD Biosciences, San Jose, CA, USA) according to the manufacturer’s instructions. Human peripheral blood mononuclear cells (PBMCs) were obtained from two healthy adult male Japanese volunteers (26 years of age) with informed consent. After the PBMCs were prepared with Ficoll, they were plated at a concentration of 1 × 10^7^ cells/mL in a 96-well flat-bottom culture plate and maintained in complete RPMI medium. They were stimulated with K-type CpG ODNs or CA-CpG for 24 h. Supernatants were subjected to ELISA to determine the levels of pan-IFN-α and IFN-γ according to the manufacturers’ instructions. All experiments using human PBMCs were approved by the Institutional Review Board of the Research Institute for Microbial Diseases, Osaka University.

### Cellular Uptake of CA-CpG and K-Type CpG ODNs

Mouse bone marrow-derived DCs (1 × 10^7^ cells/mL in a 96-well flat-bottom culture plate) were treated with Alexa 488-labeled K-type CpG ODNs (1.25 µg CpG ODNs/mL) or CA nanoparticles containing Alexa 488-labeled K-type CpG ODNs (CA-Alexa 488-CpG; 1.25 µg CpG ODNs/mL) for 10, 30, 90, 120, or 180 min. Cells were stained with 0.4 w/v% trypan blue solution (Wako, Osaka, Japan) to quench any Alexa 488-labeled K-type CpG ODNs bound to the cell surface, and then the cells were analyzed by means of flow cytometry (NovoCyte Flow Cytometer, ACEA Bioscience, San Diego, CA, USA). To separate various subsets of DCs from the collected cells, we incubated the cells with anti-mouse CD16/CD32 antibody (TONBO biosciences, San Diego, CA, USA), anti-mouse B220 antibody (BioLegend), anti-CD11c antibody (BioLegend), and anti-CD11b antibody (BioLegend) in the absence of trypan blue. In this way, the DCs were separated into the following subsets: B220^+^ CD11c^+^ plasmacytoid DCs, CD11b^+^ CD11c^+^ conventional DCs, and CD11b^−^ CD11c^+^ conventional DCs.

### Biodistribution of CpG ODNs *In Vivo*

C57BL/6J mice were treated with saline, Alexa 488-labeled K-type CpG ODNs (8 or 40 µg CpG ODNs/mouse), or CA-Alexa 488-CpG (8 µg CpG ODNs/mouse) into the ear pinna. Twenty-four hours after administration, draining lymph nodes were collected. To prepare single-cell suspensions, we incubated the draining lymph nodes with 5 µg/mL collagenase D (Wako, Tokyo, Japan) for 60 min at 37°C. We used flow cytometry to analyze the uptake of CpG ODNs by cells in draining lymph nodes.

### *In Vivo* Activation of Lymph Node Cells

C57BL/6J mice were treated with K-type CpG ODNs (8 or 40 µg CpG ODNs/mouse) or CA-CpG (8 µg CpG ODNs/mouse) into the ear pinna. Twenty-four hours after administration, draining lymph nodes were collected. To prepare single-cell suspensions, we incubated the draining lymph nodes with collagenase D (5 µg/mL) for 60 min at 37°C. Prepared cells were incubated for 8 h, culture supernatants were collected, and cytokines were measured by means of ELISA.

### Immunization

C57BL/6J mice were treated with OVA (100 µg/mouse) without or with K-type CpG ODNs (10 or 50 µg CpG ODNs/mouse) or OVA with CA-CpG (10 µg CpG/mouse) subcutaneously at the base of the tail at days 0 and 14. Using hematocrit capillary tubes (Terumo, Tokyo, Japan), we obtained blood samples from the retro-orbital venous plexus at day 21 and centrifuged the samples at 3,000 × *g* at 4°C. The resulting plasma was stored at −80°C until analysis. To investigate the adjuvant effects of CA-CpG administered by another route, we treated C57BL/6J mice with OVA (80 µg/mouse) without or with K-type CpG ODNs (8 or 40 µg CpG ODNs/mouse) or OVA with CA-CpG (8 µg CpG/mouse) into the ear pinna at days 0 and 7, and we obtained blood samples at day 14.

### Detection of OVA-Specific Antibodies

The levels of OVA-specific antibodies in plasma were determined by means of ELISA. To detect OVA-specific IgG, IgG1, and IgG2c, we coated ELISA plates (Corning, Corning, NY, USA) with OVA in carbonate buffer (10 µg/mL) overnight at 4°C. The coated plates were incubated with PBS containing 10% FCS. Plasma samples were diluted with PBS containing 10% FCS, and the dilutions were added to the OVA-coated plates. After incubation with plasma for 2 h, the coated plates were incubated with an HRP-conjugated goat anti-mouse IgG, IgG1, or IgG2c solution for 1 h at room temperature. After incubation, the color was developed with tetramethyl benzidine (Moss, Pasadena, MD, USA), the reaction was stopped with 2 N H_2_SO_4_, and the OD_450–570_ was measured on a microplate reader (Power Wave HT, BioTek, Winooski, VT, USA).

### Detection of OVA-Specific CD8^+^ CTL Induction

C57BL/6J mice were treated with OVA (80 µg/mouse) without or with K-type CpG ODNs (8 or 40 µg CpG ODNs/mouse) or CA-CpG (8 µg CpG ODNs/mouse) into the ear pinna. Draining lymph nodes were collected at day 7, and lymph node cells were prepared for determination of OVA_257–264_-specific CD8^+^ CTL responses. Lymph node cells (1 × 10^6^ cells) were added to the wells of a 96 half-well plate and then stimulated with OVA_257–264_ peptide (SIINFEKL, final concentration 5 µg/mL) for 24 h at 37°C. After the incubation, the concentration of IFN-γ in the supernatants was analyzed by means of ELISA. For the tetramer assay, lymph node cells (1 × 10^6^ cells) were stained with purified anti-mouse CD16/CD32 antibody and then with the phycoerythrin-labeled H-2K^b^/OVA_257–264_ tetramer (MBL, Aichi, Japan). Fluorescein isothiocyanate-conjugated CD8α (Abcam, Cambridge, UK), allophycocyanin-conjugated CD44 (BioLegend), and 7-amino-actinomycin D (BioLegend) were then added. The number of CD8α^+^ CD44^+^ tetramer^+^ cells was determined by flow cytometry.

### Vaccine Against Influenza Virus

C57BL/6J mice were treated with ether-treated hemagglutinin antigen-enriched virion-free split vaccine (SV; strain A/California/7/2009, H1N1, 0.5 µg/mouse) without or with K-type CpG ODNs (10 or 50 µg CpG ODNs/mouse), CA-CpG (10 µg CpG ODNs/mouse), CA nanoparticles, or alum at days 0 and 14. At day 21, we obtained blood samples from the retro-orbital venous plexus and centrifuged the samples at 3,000 × *g* at 4°C; the resulting plasma was stored at −80°C until analysis. To detect SV-specific IgG, IgG1, and IgG2c, we coated ELISA plates with SV in carbonate buffer (10 µg/mL) overnight at 4°C. The coated plates were incubated with PBS containing 10% FCS. Plasma samples were diluted with PBS containing 10% FCS, and the dilutions were added to the SV-coated plates. After incubation with plasma for 2 h, the coated plates were incubated with a solution of HRP-conjugated goat anti-mouse IgG, IgG1, or IgG2c for 1 h at room temperature. After incubation, the color was developed with tetramethyl benzidine, the reaction was stopped with 2 N H_2_SO_4_, and OD_450–570_ was measured on a microplate reader. For analysis of hemagglutination inhibition (HI) titers, plasma samples were incubated with RDE (II) (DENKA SEIKEN, Tokyo, Japan) for 18 h at 37°C and then heated at 56°C for 30 min to deactivate the enzyme. To avoid nonspecific hemagglutination, plasma samples were treated with guinea pig red blood cells for 1 h at room temperature, and the supernatants were serially diluted 2-fold in microtiter plates. An equal volume of influenza virus (strain A/California/7/2009, H1N1) was added to each well, and the plates were incubated for 1 h at room temperature. Guinea pig red blood cells were added and allowed to settle for 1 h at 4°C. The HI titer was determined from the dilution of the last well that contained non-agglutinated guinea pig red blood cells. Moreover, immunized mice were challenged intranasally with 7.2 × 10^3^ pfu (10-LD50) of influenza virus (strain A/PR/8/34, H1N1) (28). Body weights and survival rates of challenged mice were monitored for 14 days. Ether-treated hemagglutinin antigen-enriched virion-free split vaccine and influenza virus were kindly provided by the Research Foundation for Microbial Diseases of Osaka University, Suita, Japan.

### Weight of Tissues

K-type CpG ODNs (10 or 50 µg CpG ODNs/mouse) or CA-CpG (10 µg CpG ODNs/mouse) was administered to C57BL/6J mice subcutaneously at the base of the tail at days 0, 2, and 4. Twenty-four hours after the final administration, the entire spleen, the entire liver, and draining lymph nodes were isolated and weighed.

### Statistical Analyses

Statistical analyses were performed with GraphPad Prism (GraphPad Software, San Diego, CA, USA). All data are presented as mean with SD or SEM. Significant differences between control groups and experimental groups were determined by means of Dunnett’s test, Tukey’s test, or Student’s *t*-test. A *p* value of <0.05 was considered to indicate statistical significance.

## Results

### CA-CpG Had High CpG ODN Encapsulation Efficiency

**Figure 1:**
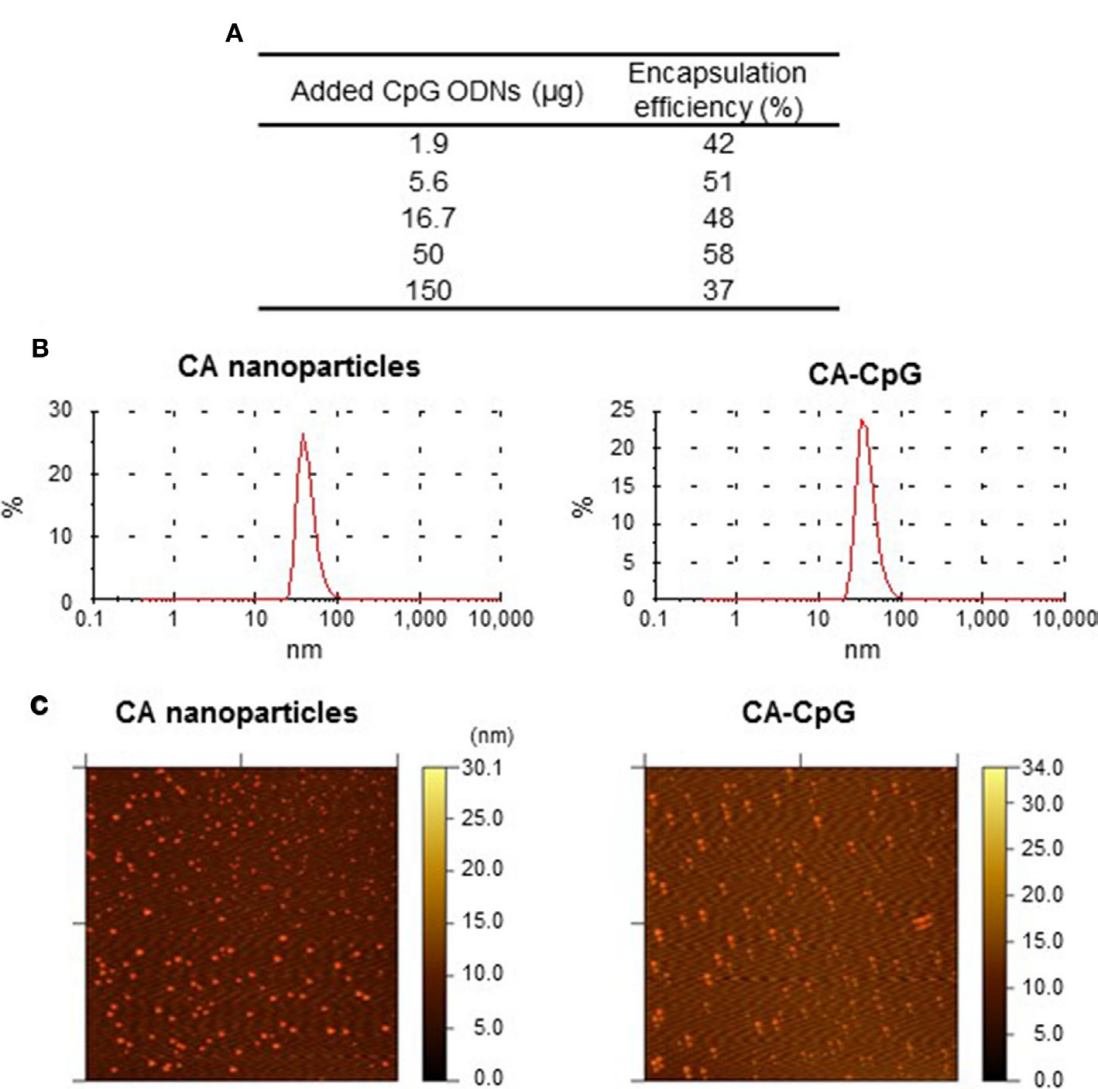
Encapsulation efficiency and size distribution of carbonate apatite (CA)-cytosine-phosphate-guanine (CpG). **(A)** The use of various amounts of K-type CpG oligodeoxynucleotides (ODNs) to generate CA-CpG showed that encapsulation efficiency was highest at 50 µg CpG ODNs. **(B,C)** The size distribution of CA-CpG was determined by dynamic light scattering **(B)** and atomic force microscopy **(C)**. CA-CpG was roughly the same size as CA nanoparticles that did not contain K-type CpG ODNs.

We evaluated the usefulness of CA nanoparticles as CpG ODN delivery vehicles. We began by determining the amount of K-type CpG ODNs encapsulated in CA-CpG. We found that the amount of K-type CpG ODNs used to generate CA-CpG affected the efficiency with which the K-type CpG ODNs was encapsulated in CA-CpG (Figure 1A). When we used 50 µg K-type CpG ODNs, the encapsulation efficiency was high (58%). Therefore, we used CA-CpG prepared with 50 µg K-type CpG ODNs for subsequent experiments. Dynamic light scattering showed that both the empty CA nanoparticles and CA-CpG were uniform and monodispersed, with hydrodynamic diameters ranging from 30 to 40 nm, respectively (Figure 1B). In addition, we confirmed that CA-Alexa 488-CpG was also uniform and monodispersed, with hydrodynamic diameters ranging from 30 to 40 nm, as was the case for both the empty CA nanoparticles and CA-CpG (data not shown). Atomic force microscopy showed that the sizes of the CA nanoparticles and CA-CpG ranged from 13 to 25 nm (Figure 1C). These results suggest that we successfully generated nanosize CA-CpG with high encapsulation efficiency for K-type CpG ODNs.

### CA-CpG Induced IFN-α Production and Enhanced the Production of Other Cytokines in Mouse DCs and Human PBMCs

**Figure 2:**
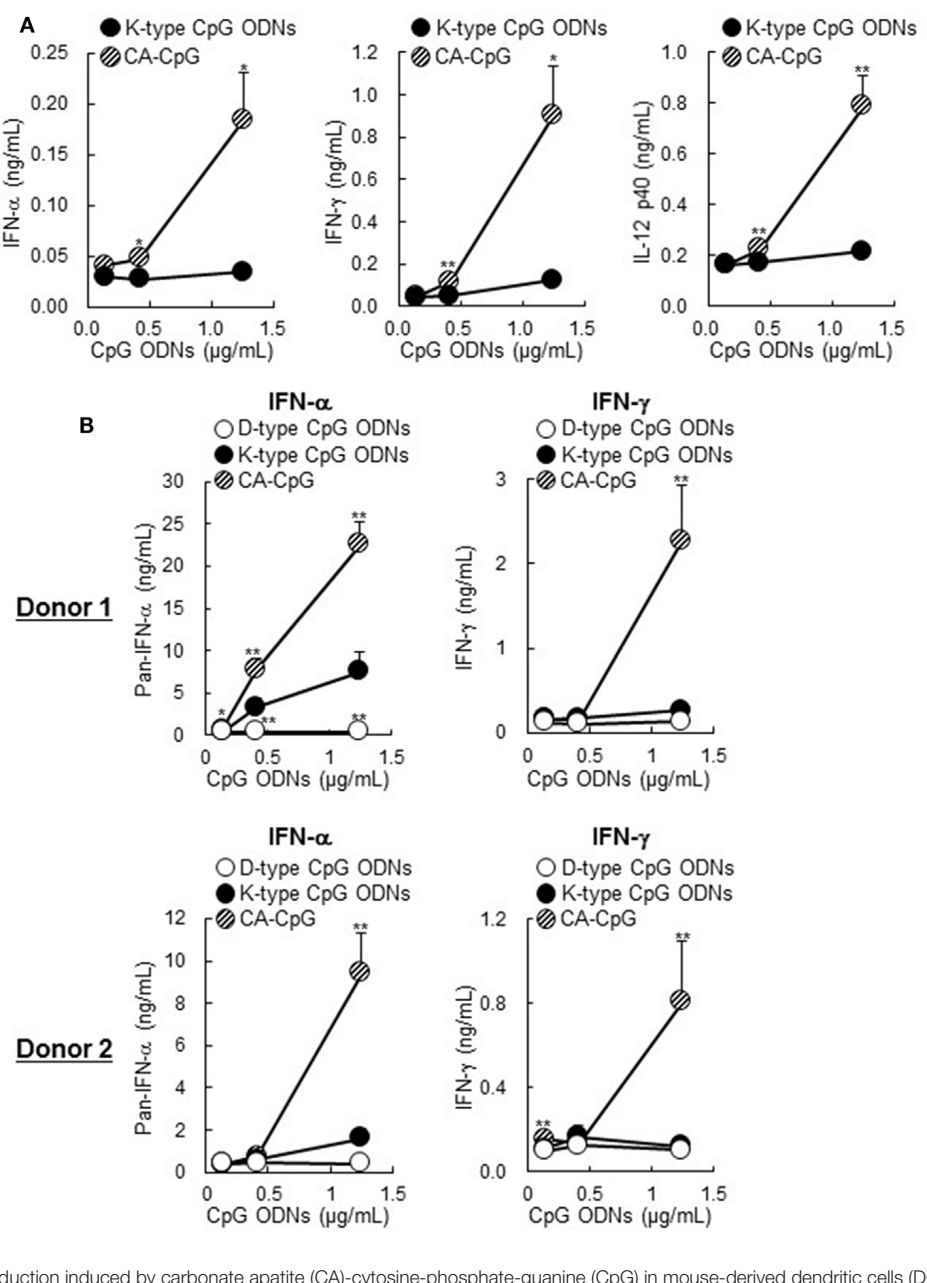
Cytokine production induced by carbonate apatite (CA)-cytosine-phosphate-guanine (CpG) in mouse-derived dendritic cells (DCs) and human peripheral blood mononuclear cells (PBMCs). Mouse-derived DCs **(A)** and human PBMCs **(B)** were treated with several concentrations (adjusted for CpG oligodeoxynucleotides (ODN) concentration) of CA-CpG, K-type CpG ODNs, or D-type CpG ODNs for 24 h, and the cytokine concentrations in the supernatants were then measured by ELISA. **(A)** In DCs, CA-CpG induced higher IFN-α, IFN-γ, and IL-12 p40 production than did K-type CpG ODNs, *n* = 4 per group. **(B)** In PBMCs, CA-CpG induced higher levels of pan-IFN-α and IFN-γ production than did K-type or D-type CpG ODNs, *n* = 5 per group. These data suggest that CA-CpG has some characteristics of D-type CpG ODNs. Data are given as mean ± SD. *p < 0.05, **p < 0.01 vs. K-type CpG ODN-treated group as indicated by Student’s *t*-test **(A)** or Dunnett’s test **(B)**.

To determine the immunostimulatory effects of CA-CpG on DCs, we treated mouse-derived DCs with CA-CpG or K-type CpG ODNs *in vitro* and measured cytokine levels in the supernatants by means of ELISA (Figure 2A). DCs stimulated with CA-CpG produced substantial amounts of not only IFN-γ and IL-12 p40 but also IFN-α, whereas K-type CpG ODNs did not induce cytokine production at the tested doses. In human PBMCs, higher levels of IFN-α and IFN-γ were produced in response to CA-CpG than in response to K-type CpG ODNs (Figure 2B). These data suggest that CA-CpG might be superior to K-type CpG ODNs as an adjuvant. In addition, they suggest that CA-CpG has some characteristics of D-type CpG ODNs, because IFN-α is known to be a D-type CpG ODN-specific cytokine.

### Pattern of CA-CpG Uptake by DCs Differed From That of K-Type CpG ODNs *In Vitro*

**Figure 3:**
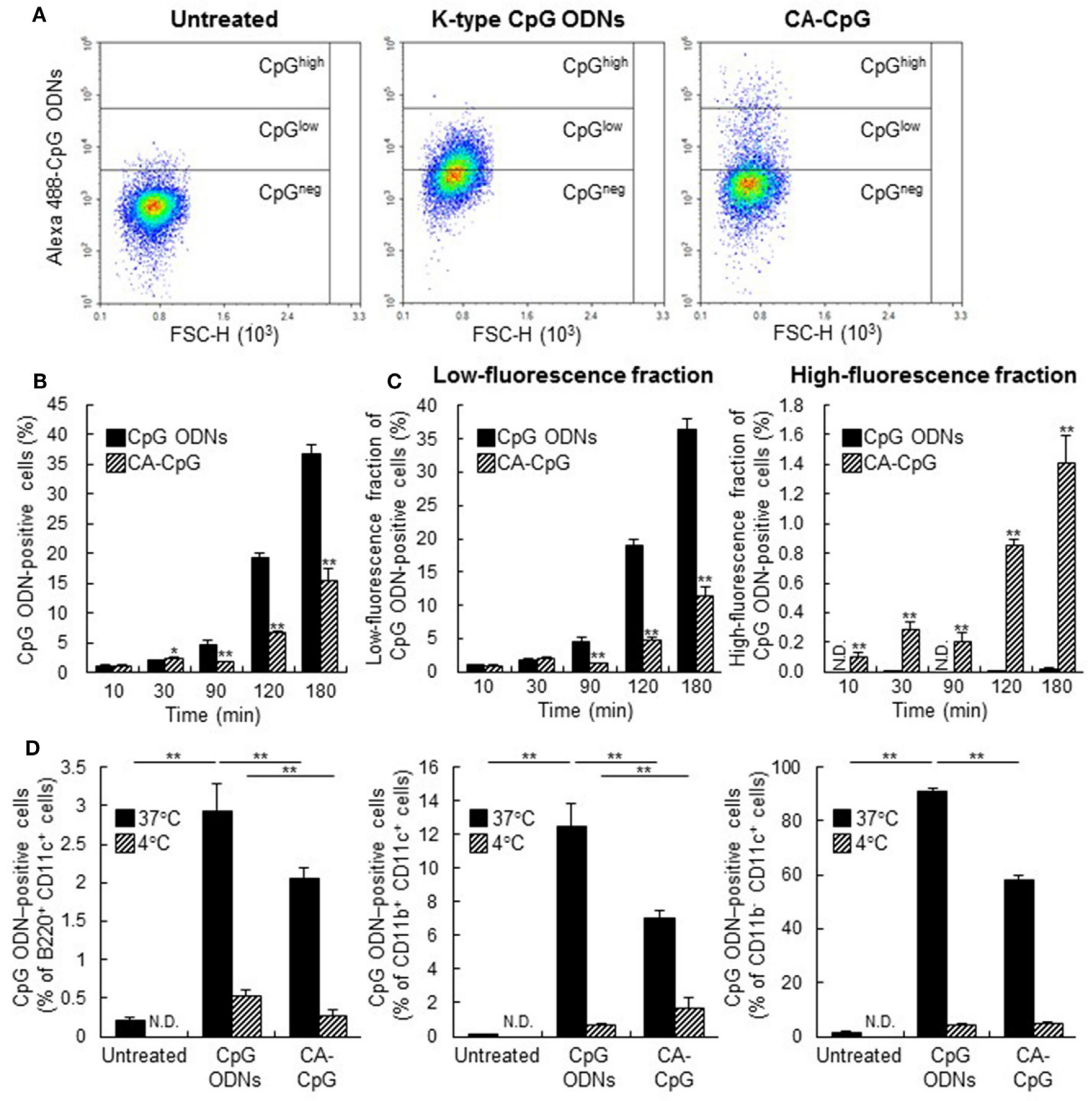
Cellular uptake of carbonate apatite (CA)-cytosine-phosphate-guanine (CpG). **(A–C)** Comparison of cellular uptake patterns of CA-CpG and K-type CpG oligodeoxynucleotides (ODNs). Mouse-derived dendritic cells (DCs) were treated with CA-Alexa 488-CpG or Alexa 488-labeled K-type CpG ODNs. At various time points after treatment, K-type CpG ODNs internalized in DCs were measured by flow cytometry after trypan blue quenching. **(A)** Representative dot plots show Alexa 488-labeled CpG ODNs in DCs vs. forward scatter (FSC-A). **(B)** Quantification of fluorescence revealed that uptake of CA-CpG into DCs was lower than uptake of K-type CpG ODNs, *n* = 5 per group. **(C)** Separation of the dot plots for the CpG ODN-positive fraction in panel **(A)** into graphs for high-fluorescence and low-fluorescence fractions revealed that the number of CpG ODNs taken up by each DC in the CA-CpG-treated groups was larger than that in the K-type CpG ODN-treated groups, *n* = 5 per group. **(D)** Subsets of DCs that took up CA-CpG. Mouse-derived DCs were treated with CA-Alexa 488-CpG or Alexa 488-labeled K-type CpG ODNs for 3 h at 4°C or 37°C. Uptake of CA-Alexa 488-CpG or Alexa 488-labeled K-type CpG ODNs into each subset of DCs was measured by flow cytometry without trypan blue quenching, *n* = 5 per group. Data are given as mean ± SD. *p < 0.05, **p < 0.01 vs. K-type CpG ODN-treated group as indicated by Student’s *t*-test **(B,C)** or Dunnett’s test **(D)**. ND, not detected.

To elucidate the mechanism of the immunostimulatory effects of CA-CpG, we compared the cellular internalization of CA-CpG and K-type CpG ODNs in mouse DCs by using Alexa 488-labeled K-type CpG ODNs. DCs were treated with CA-Alexa 488-CpG or with Alexa 488-labeled K-type CpG ODNs, and cells were stained with trypan blue solution to quench any Alexa 488-labeled K-type CpG ODNs bound to the cell surface, so that only internalized K-type CpG ODNs were detected during flow cytometry (Figures 3A–C). The amount of Alexa 488-labeled K-type CpG ODNs in DCs treated with CA-Alexa 488-CpG or Alexa 488-labeled K-type CpG ODNs increased over time, and at 90, 120, and 180 min after treatment the groups treated with Alexa 488-labeled K-type CpG ODNs had more cells that were positive for K-type CpG ODNs than did the groups treated with CA-Alexa 488-CpG (Figures 3A,B). For more precise analysis, the fractions that were positive for CpG ODNs were divided into two fractions, one with low fluorescence and one with high fluorescence; then we again compared the groups treated with CA-Alexa 488-CpG and the groups treated with Alexa 488-labeled K-type CpG ODNs (Figures 3A,C). In the low-fluorescence fraction, at 90, 120, and 180 min after treatment, the groups treated with Alexa 488-labeled K-type CpG ODNs had more cells that were positive for K-type CpG ODNs than did the CA-Alexa 488-CpG-treated groups. In contrast, in the high-fluorescence fraction at all time points, the CA-Alexa 488-CpG-treated groups had significantly more cells that were positive for K-type CpG ODNs than did the groups treated with Alexa 488-labeled K-type CpG ODNs. On the basis of these results, we speculated that certain subsets of DCs might have taken up CA-CpG more efficiently than others. To evaluate this possibility, we categorized DCs into the following subsets: B220^+^ CD11c^+^ plasmacytoid DCs, CD11b^+^ CD11c^+^ conventional DCs, and CD11b^−^ CD11c^+^ conventional DCs. Then we analyzed the CpG ODN-positive cells in each subset at both 37°C and 4°C, so that we could evaluate nonspecific binding of CA-Alexa 488-CpG or Alexa 488-labeled K-type CpG ODNs to the cell surface (Figure 3D). There were fewer CpG ODN-positive cells at 4°C than at 37°C, indicating that the binding of CA-CpG or K-type CpG ODNs on the cell surface could be ignored in this assay. At 37°C, in all the subsets of DCs, the number of CpG ODN-positive cells in the CA-CpG-treated groups was lower than that in the K-type CpG ODN-treated groups (Figure 3D), indicating that CA-CpG was taken up into all the subsets of DCs equally, as was the case for the K-type CpG ODNs.

### CA-CpG Enhanced Cytokine Production *In Vivo*

**Figure 4:**
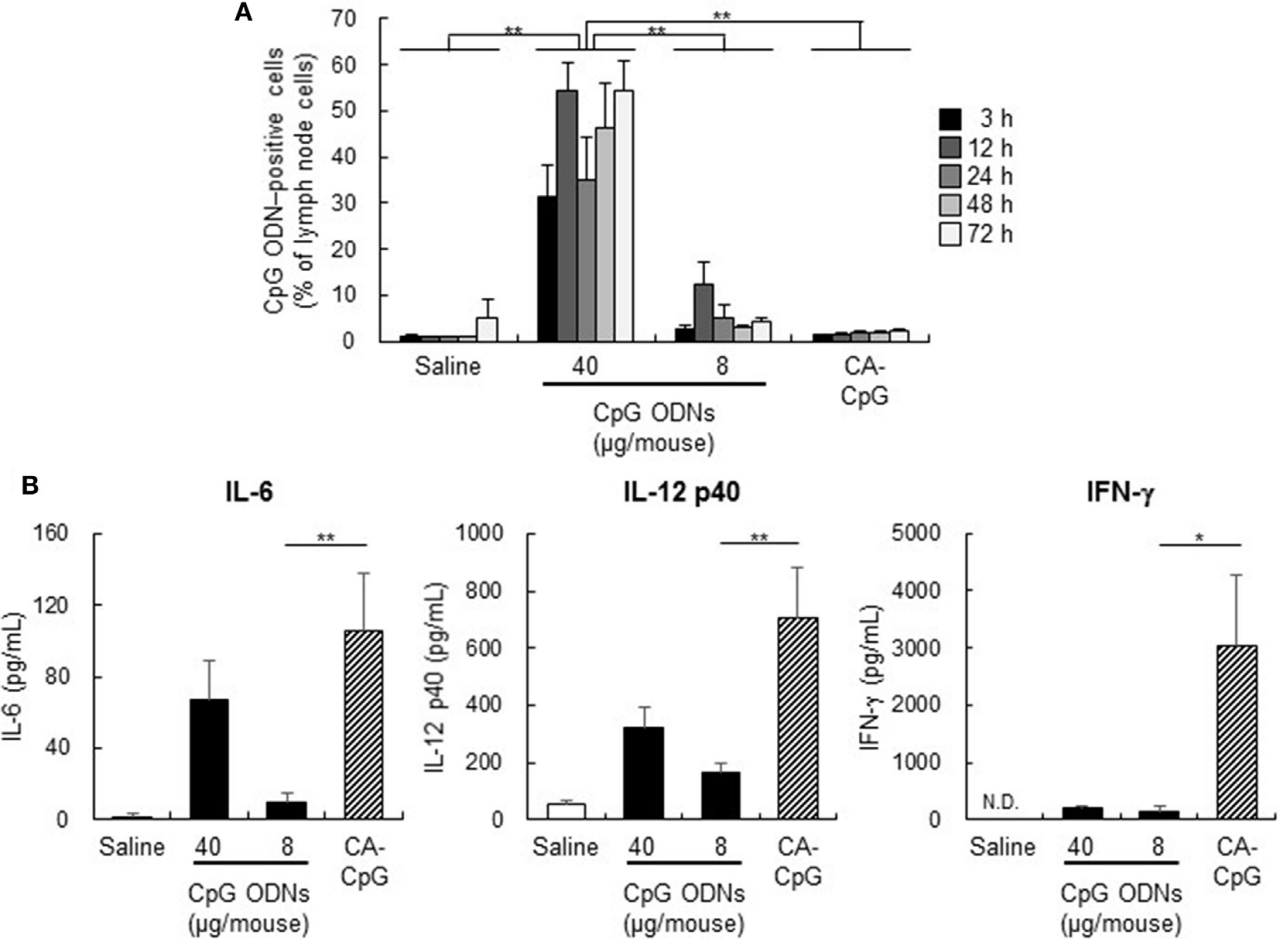
Biodistribution of carbonate apatite (CA)-cytosine-phosphate-guanine (CpG) *in vivo* and cytokine production induced by CA-CpG in draining lymph nodes. **(A)** Biodistribution of CA-CpG *in vivo*. Mice were treated with CA-Alexa 488-CpG or Alexa 488-labeled K-type CpG oligodeoxynucleotides (ODNs) into the ear pinna, and internalization of K-type CpG ODNs by cells in draining lymph nodes was measured by flow cytometry at the indicated time points after trypan blue quenching. Treatment with CA-CpG did not increase the number of cells that internalized CpG ODNs, *n* = 5 per group. **(B)** Cytokine production induced by CA-CpG in draining lymph nodes. CA-CpG (8 µg CpG ODNs/mouse) or K-type CpG ODNs (8 or 40 µg CpG ODNs/mouse) were administered into the ear pinna. After 24 h, lymph node cells in draining lymph nodes were harvested and cultured for 8 h, and the levels of IL-6, IL-12 p40 and IFN-γ in the culture supernatants were evaluated. CA-CpG induced higher levels of cytokine production than did K-type CpG ODNs, *n* = 5 per group. Data are given as mean ± SEM. *p < 0.05, **p < 0.01 as indicated by Tukey’s test **(A)** or vs. K-type CpG ODNs (8 µg CpG ODNs/mouse)-treated group as indicated by Dunnett’s test **(B)**. ND, not detected.

Generally, the delivery of an adjuvant to lymph nodes, and specifically to DCs in lymph nodes, is important for the efficient induction of adaptive immunity. However, the efficiency with which CA-CpG delivers CpG ODNs to DCs in lymph nodes is unknown. Therefore, to assess the distribution and uptake of CA-CpG into lymph nodes, we measured the quantity of CpG ODN-positive cells in draining lymph nodes by flow cytometry after administration of CA-Alexa 488-CpG or Alexa 488-labeled K-type CpG ODNs to mice (Figure 4A). Whereas in the group treated with Alexa 488-labeled K-type CpG ODNs at 40 µg CpG ODNs/mouse (the positive control), large proportions of the lymph node cells were CpG ODN-positive, there were no significant differences between the proportions of CpG ODN-positive cells in the groups treated with CA-Alexa 488-CpG at 8 µg CpG ODNs/mouse and the groups treated with Alexa 488-labeled K-type CpG ODNs at 8 µg CpG ODNs/mouse at any time point. These results suggest that the CA nanoparticles did not increase the number of CpG ODNs taken up by DCs in lymph nodes.

Next, to determine the immunostimulatory effects of CA-CpG *in vivo*, we measured cytokine production in draining lymph nodes after CA-CpG administration (Figure 4B). We found that production of the cytokines IL-6, IL-12 p40, and IFN-γ in draining lymph nodes was substantially higher after treatment with CA-CpG than after treatment with K-type CpG ODNs (Figure 4B). These data suggest that CA-CpG markedly enhanced innate immune activation responses, such as cytokine production, relative to K-type CpG ODNs both *in vitro* and *in vivo*.

### CA-CpG Enhanced Antigen-Specific Antibody Responses and CD8^+^ CTL Responses *In Vivo*

**Figure 5:**
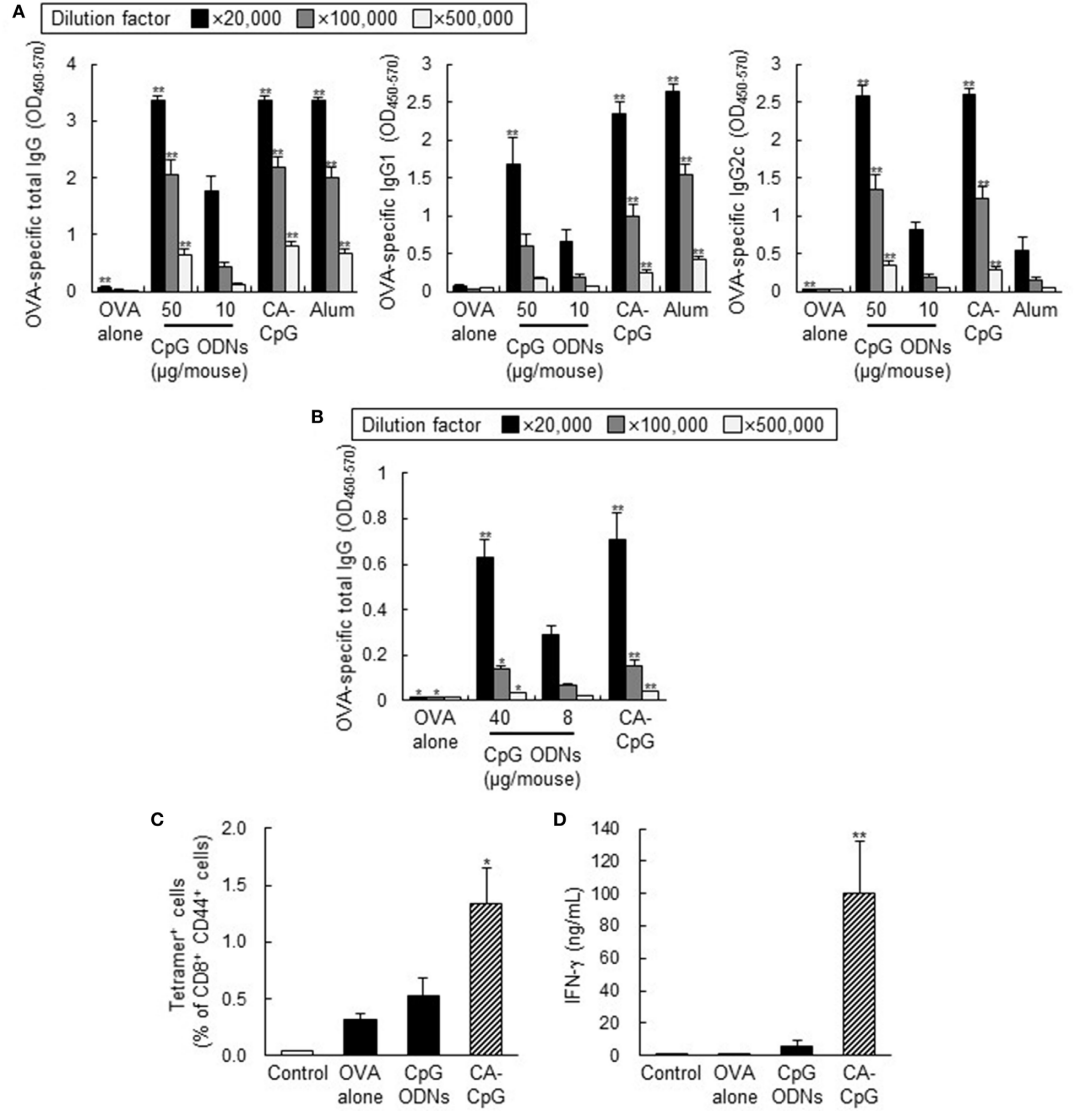
Adjuvant effects of carbonate apatite (CA)-cytosine-phosphate-guanine (CpG) in mice. Mice were immunized subcutaneously at the base of the tail **(A)** or into the ear pinna **(B–D)** with ovalbumin (OVA) plus CA-CpG at 10 µg CpG oligodeoxynucleotides (ODNs)/mouse **(A)** or 8 µg CpG ODNs/mouse **(B–D)**; with OVA plus K-type CpG ODNs at 10 or 50 µg CpG ODNs/mouse **(A)**, at 8 or 40 µg CpG ODNs/mouse **(B)**, or at 8 µg CpG ODNs/mouse **(C,D)**; or with OVA plus alum. **(A,B)** OVA-specific antibody responses. Levels of OVA-specific total IgG, IgG1, and IgG2c in plasma were evaluated by ELISA 7 days after the final immunization, *n* = 5 per group. **(C,D)** CD8^+^ cytotoxic T lymphocyte (CTL) responses. OVA_257–264_-specific CD8^+^ CTL responses were monitored by means of a tetramer assay in untreated mice and in mice immunized with OVA alone, with OVA plus K-type CpG ODNs, or with CA-CpG **(C)**, *n* = 5 per group. Lymph node cells were stimulated with OVA_257–264_ peptide, and the concentration of IFN-γ in supernatants was analyzed by ELISA **(D)**, *n* = 5 per group. The data in this figure suggest that a simple mixture containing CA-CpG and an antigen-induced antigen-specific antibody responses and CD8^+^ CTL responses that were superior to those induced by K-type CpG ODNs and alum, thus demonstrating the potential of CA-CpG as a vaccine adjuvant. Data are given as mean ± SEM. *p < 0.05, **p < 0.01 vs. K-type CpG ODNs (10 or 8 µg CpG ODNs/mouse)-treated group as indicated by Dunnett’s test.

To evaluate the *in vivo* effects of the use of CA-CpG as a vaccine adjuvant in mice, we assessed antibody responses and CD8^+^ CTL responses following co-administration of CA-CpG or K-type CpG ODNs with OVA, a model protein antigen. Mice were immunized with OVA plus CA-CpG (10 µg CpG ODNs/mouse) or OVA plus K-type CpG ODNs (10 or 50 µg CpG ODNs/mouse) subcutaneously at the base of the tail. As a positive control, we used alum, which is widely used throughout the world as an adjuvant for human vaccines. Seven days after the final immunization, the levels of OVA-specific total IgG, IgG1, and IgG2c antibodies in plasma were analyzed by ELISA (Figure 5A). Levels of antigen-specific IgG subclasses reflect the subset of CD4^+^ T cells that are induced by vaccination, with production of IgG1 and IgG2c corresponding to Th2- and Th1-type responses, respectively. Although mice immunized with OVA plus alum showed high levels of total IgG and IgG1 production, mice immunized with OVA plus CA-CpG or OVA plus K-type CpG ODNs produced high levels not only of total IgG and IgG1 but also of IgG2c. Mice immunized with OVA plus CA-CpG (10 µg CpG ODNs/mouse) produced significantly higher levels of OVA-specific total IgG, IgG1, and IgG2c than did mice immunized with OVA plus K-type CpG ODNs (10 µg CpG ODNs/mouse). Antibody production in groups treated with OVA plus CA-CpG (10 µg CpG ODNs/mouse) was comparable to that in groups treated with OVA plus K-type CpG ODNs (50 µg CpG ODNs/mouse).

Next, to evaluate the adjuvant effects of CA-CpG when administered by a different route, we immunized mice with OVA plus CA-CpG or OVA plus K-type CpG ODNs into the ear pinna and then measured OVA-specific antibody responses (Figure 5B). As was the case for immunization subcutaneously at the base of the tail, the level of OVA-specific total IgG induced by immunization with OVA plus CA-CpG (8 µg CpG ODNs/mouse) was significantly higher than the level induced by immunization with OVA plus K-type CpG ODNs (8 µg CpG ODNs/mouse) and was comparable to the level induced by administration of OVA plus K-type CpG ODNs (40 µg CpG ODNs/mouse).

Clearance of viruses is known to require not only antibody responses but also strong CD8^+^ CTL responses, which are characterized by IFN-γ production by CD8^+^ T cells. To investigate the ability of CA-CpG to induce OVA-specific CD8^+^ CTL responses, we vaccinated mice with OVA plus CA-CpG and then examined the frequency of H-2K^b^/OVA_257–264_ tetramer^+^ CD8^+^ T cells (Figure 5C) and measured the production of H-2K^b^/OVA_257–264_-specific IFN-γ (Figure 5D). The frequency of H-2K^b^/OVA_257–264_ tetramer^+^ CD8^+^ T cells induced by OVA plus CA-CpG was significantly higher than the frequency induced by OVA plus K-type CpG ODNs (Figure 5C). Furthermore, the production of OVA_257–264_-specific IFN-γ induced by OVA plus CA-CpG was significantly higher than that induced by immunization with OVA plus K-type CpG ODNs (Figure 5D). Taken together, these results suggest that, *in vivo*, CA-CpG stimulated antigen-specific antibody responses and CD8^+^ CTL responses more effectively than did K-type CpG ODNs.

### CA-CpG Showed Strong Adjuvant Effects and Preventive Effects in Murine Influenza Models

**Figure 6:**
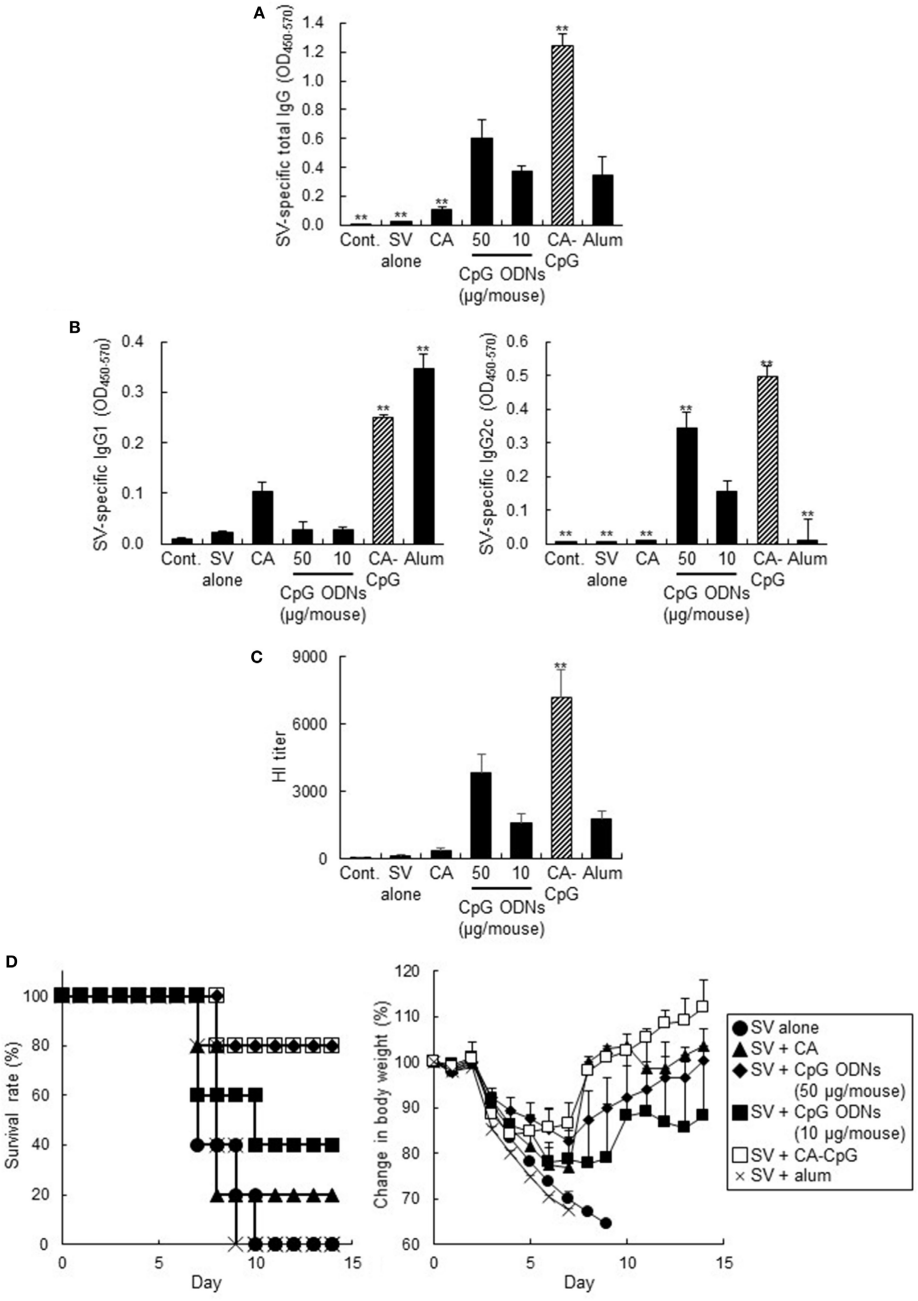
Vaccine adjuvant effect of carbonate apatite (CA)-cytosine-phosphate-guanine (CpG) against influenza virus. Mice were immunized subcutaneously at the base of the tail with SV alone or with SV plus CA nanoparticles that did not contain CpG oligodeoxynucleotides (ODNs), with SV plus K-type CpG ODNs (10 or 50 µg CpG/mouse), with SV plus CA-CpG (10 µg CpG/mouse), or with SV plus alum. **(A,B)** SV-specific antibody responses. Levels of SV-specific total IgG **(A)**, IgG1 **(B)**, and IgG2c **(B)** in plasma were evaluated by ELISA 7 days after the final immunization. **(C)** Hemagglutination inhibition (HI) assay. HI titers in plasma samples were evaluated 7 days after the final immunization. **(D)** Preventive effects against influenza virus. Fourteen days after the final immunization, mice were challenged with a 10-LD_50_ dose of influenza virus A/PR/8 (H1N1). Body weights and survival rates were monitored for the next 14 days. The data in this figure suggest that CA-CpG acted as an influenza vaccine adjuvant in mice. *n* = 5 per group. Data are given as mean ± SEM. **p < 0.01 vs. K-type CpG ODNs (10 µg CpG ODNs/mouse)-treated group as indicated by Dunnett’s test.

We examined the vaccine adjuvant effects of CA-CpG by using clinically relevant influenza vaccination models in mice. Mice were immunized with A/California/7/2009 (H1N1) SV plus CA-CpG (10 µg CpG ODNs/mouse), K-type CpG ODNs (10 or 50 µg/mouse), CA nanoparticles, or Alum. The levels of SV-specific total IgG, IgG1, and IgG2c antibodies in plasma were analyzed by ELISA after the final immunization (Figures 6A,B). Mice immunized with SV plus CA-CpG (10 µg CpG ODNs/mouse) showed significantly higher levels of SV-specific total IgG, IgG1, and IgG2c than did mice immunized with OVA plus K-type CpG ODNs (10 and 50 µg CpG ODNs/mouse). The levels of SV-specific total IgG and IgG2c in mice treated with SV plus CA-CpG were significantly higher than those in mice treated with SV plus alum, whereas there was no difference in SV-specific IgG1 levels between these two groups of mice. The levels of total IgG and IgG1 in mice treated with SV plus CA nanoparticles were higher than the levels in mice treated with SV alone but were significantly lower than the levels in CA-CpG-treated mice. Next, we examined the neutralizing potential of these antibodies by using the HI assay against influenza virus A/California/7/2009 (Figure 6C), because this assay is widely used to evaluate anti-influenza virus neutralizing and protective antibodies. The HI titers showed the same trends as the levels of SV-specific total IgG, and mice immunized with SV plus CA-CpG had significantly higher HI titers than did mice immunized with SV plus K-type CpG ODNs (10 µg CpG ODNs/mouse). After the final immunization, we challenged the immunized mice with mouse-adapted influenza virus A/PR/8/34 (H1N1), which is a heterologous influenza virus from the one used as a vaccine antigen. Weights and survival rates of the infected mice were observed every day for 2 weeks (Figure 6D). Much greater pathogenesis was observed in the mice immunized without an adjuvant or with alum than in the mice co-immunized with CA-CpG or K-type CpG ODNs (10 or 50 µg CpG ODNs/mouse), as indicated by survival rate and weight loss. All mice died within 10 days after viral infection in the groups that received SV alone or SV plus alum, and 80% of the mice immunized with SV plus CA nanoparticles died within 7 days. Although only 40% of the mice immunized with SV plus K-type CpG ODNs (10 µg CpG ODNs/mouse) survived 14 days after challenge, 80% of mice immunized with SV plus CA-CpG (10 µg CpG ODNs/mouse) or K-type CpG ODNs (50 µg/mouse) survived, and they regained body weight more rapidly. These results indicate that CA-CpG is a potent vaccine adjuvant that protects against viral infection better than K-type CpG ODNs or alum.

### Adjuvant Effects of CA-CpG Depended on Both IFN-α and IL-12

**Figure 7:**
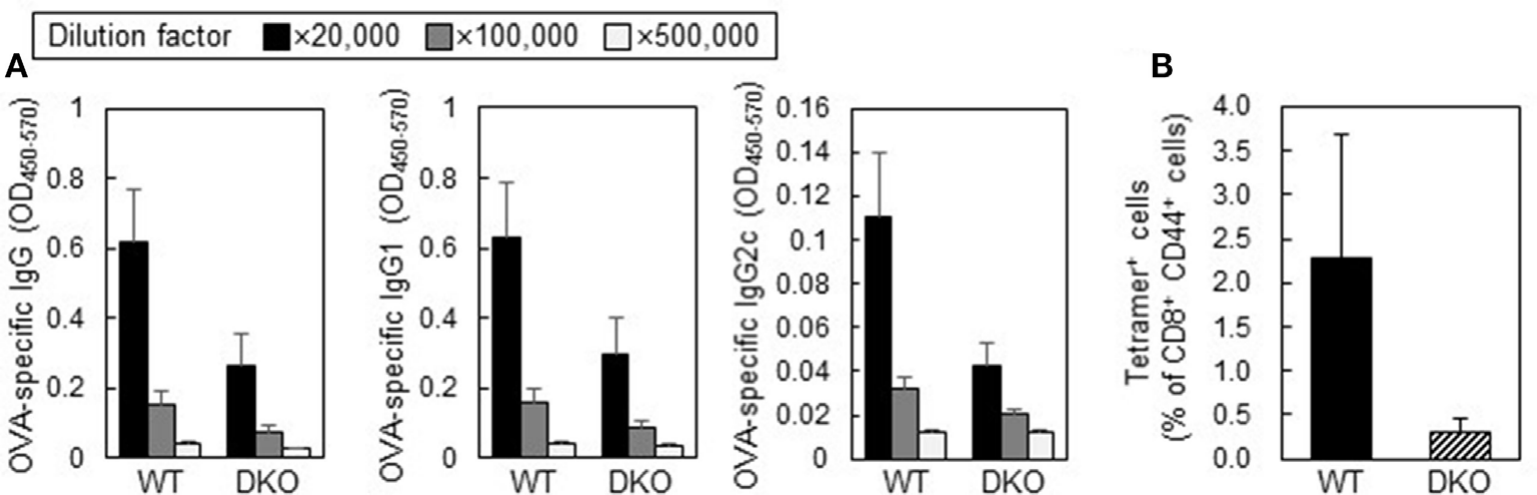
Adjuvant activities of carbonate apatite (CA)-cytosine-phosphate-guanine (CpG) in *IFN-*α*/*β* receptor 2 (Ifnar2)* and *Il-12 p40* double-deficient mice (DKO mice). Wild-type (WT) mice or DKO mice were immunized subcutaneously at the base of the tail with ovalbumin (OVA) plus CA-CpG (10 µg CpG/mouse) at days 0 and 14. **(A)** OVA-specific antibody responses. Levels of OVA-specific total IgG, IgG1, and IgG2c in plasma were evaluated by ELISA 7 days after the final immunization, *n* = 5 per WT group and *n* = 4 per DKO group. **(B)** CD8^+^ cytotoxic T lymphocyte (CTL) responses. OVA_257–264_-specific CD8^+^ CTL responses were monitored in mice by tetramer assay 7 days after the final immunization, *n* = 5 per WT group and *n* = 4 per DKO group.

Activation of CD8^+^ CTL responses requires T-cell activation by DCs via costimulatory signals and cytokine-mediated signals. For example, IFN-α and IL-12 work as critical survival signals that expand and differentiate effector CD8^+^ T cells (29–31). Therefore, to elucidate the involvement of IFN-α and IL-12 in antibody responses and CD8^+^ CTL responses induced by CA-CpG, we treated mice deficient in both *Ifnar2* and *Il-12 p40* with OVA plus CA-CpG (Figure 7). The levels of OVA-specific total IgG, IgG1, and IgG2c in these double-deficient mice immunized with OVA plus CA-CpG tended to be lower than the levels in wild-type mice (Figure 7A). In addition, the frequency of H-2K^b^/OVA_257–264_ tetramer^+^ CD8^+^ T cells in the double-deficient mice tended to be lower than that in the wild-type mice (Figure 7B). These results suggest that CA-CpG enhanced both Th1-type humoral and cellular immune responses *via* the IFN-α pathway, the IL-12 pathway, or both.

### Adverse Effects of CpG ODNs Were Not Enhanced by CA-CpG

**Figure 8:**
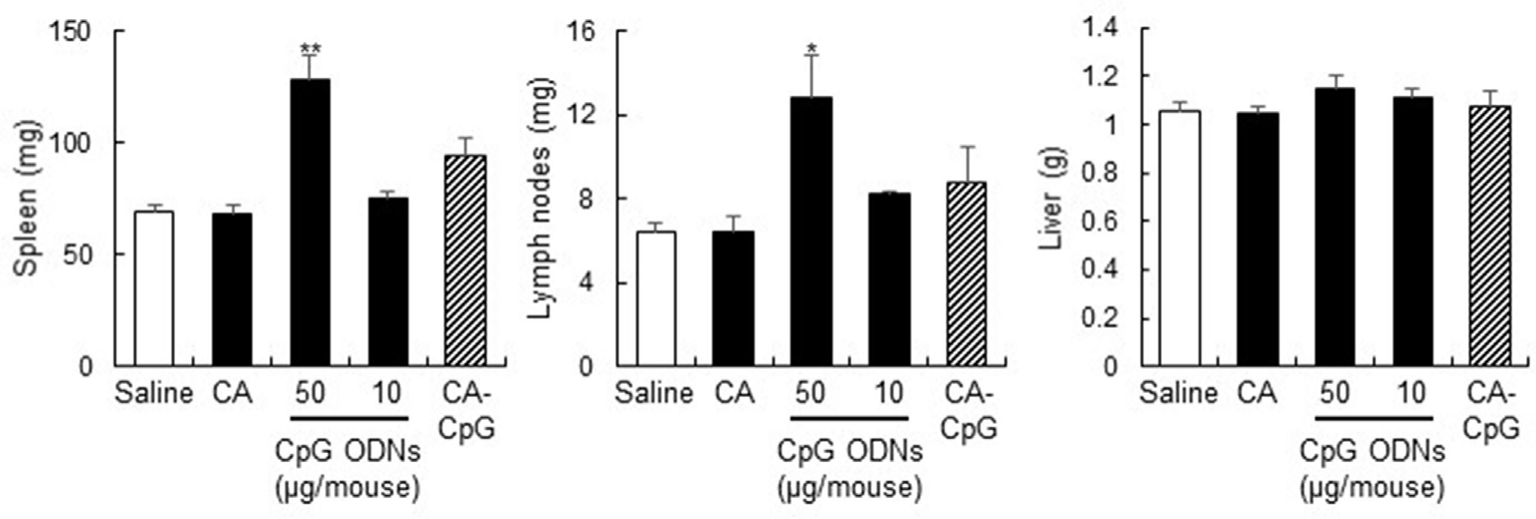
Strong adjuvant effects of carbonate apatite (CA)-cytosine-phosphate-guanine (CpG) without splenomegaly. Mice were treated with CA-CpG (10 µg CpG oligodeoxynucleotides (ODNs)/mouse), CA nanoparticles or K-type CpG ODNs (10 or 50 µg CpG ODNs/mouse) subcutaneously at the base of the tail at days 0, 2, and 4. Twenty-four hours after the final administration, the spleen, liver, and lymph nodes were weighed. CA-CpG did not induce splenomegaly; in contrast, a high dose of K-type CpG ODNs did induce splenomegaly, *n* = 5 per group. Data are given as mean ± SEM. *p < 0.05, **p < 0.01 vs. saline-treated group as indicated by Tukey’s test.

Cytosine-phosphate-guanine ODNs are known to cause splenomegaly and to disrupt splenic microarchitecture in mice after repeated administration (32). To assess the adverse effects of CA-CpG, we treated mice three times every other day with CA-CpG or K-type CpG ODNs and then measured tissue weights (Figure 8). No significant differences in liver weight were observed between the groups. In contrast, spleen weights and draining lymph node weights in mice treated with K-type CpG ODNs at 50 µg CpG ODNs/mouse were higher than those in mice treated with CA-CpG (10 µg CpG ODNs/mouse), although the difference was statistically significant only for spleen weights. The effects of CA-CpG (10 µg CpG ODNs/mouse) were almost the same as those of K-type CpG ODNs at 10 µg CpG ODNs/mouse. These results suggest that CA-CpG improved the efficacy of CpG ODNs without enhancing their adverse effects.

## Discussion

Because systemic dissemination of CpG ODNs away from the site of injection occurs rapidly after administration, high doses of CpG ODNs are required for induction of vaccine effects, thus increasing the risk of adverse events (33). Therefore, the development of easy to fabricate, high encapsulation capacity CpG ODN delivery vehicles that enhance the adjuvant activity of CpG ODNs and reduce their adverse effects is necessary. In addition, after cellular uptake, the delivery vehicle must degrade rapidly *via* an endosomal or lysosomal processing pathway so that the CpG ODNs can bind to endosomal TLR9. Several types of particle-based delivery vehicles have been reported, including cationized gelatin nanoparticles (34), calcium phosphate nanoparticles (35), poly(lactic-*co*-glycolic acid) nanoparticles (36, 37), Ficoll nanoparticles (38), poly(propylene sulfide) nanoparticles (39), and liposomes (40, 41). However, there have been no reports suggesting that these particles can enhance the adjuvant activity of K-type CpG ODNs by giving them the ability to induce IFN-α production *in vivo*. We showed here that CA nanoparticles can serve as K-type CpG ODN delivery vehicles and that they enhance the adjuvant activity of K-type CpG ODNs by inducing IFN-α production both *in vitro* and *in vivo*.

Carbonate apatite nanoparticles have been shown to efficiently encapsulate siRNA (26). In this study, we showed that CA nanoparticles can also encapsulate K-type CpG ODNs, with an encapsulation efficiency of about 60% (Figure 1A). Researchers have tried to enhance the adjuvant activity of CpG ODNs by encapsulating them in microparticles and nanoparticles, but the encapsulation efficiency has been very low (42–44). Because CpG ODNs are negatively charged, cationic materials such as protamine and chitosan have been used to bind CpG ODNs and facilitate their incorporation into particles. For example, Fisher et al. showed that CpG ODNs could be encapsulated with approximately 90% efficiency into protamine-coated poly(lactic-*co*-glycolic acid) particles, whereas the encapsulation efficiency was only 8% for bare particles (43). Peine et al. (44) and Zhang et al. (42) showed that CpG ODNs could be encapsulated with 30% efficiency into acetalated dextran particles and poly(lactic-*co*-glycolic acid) particles, respectively, without the use of cationic materials, by optimization of the conditions used to generate the particles. These results suggest that encapsulation of CpG ODNs into CA nanoparticles with relatively high efficiency can be achieved without the use of cationic materials.

Here, we showed that, compared with K-type CpG ODNs, CA-CpG induced higher levels of cytokine production in mouse DCs and human PBMCs (Figure 2). Notably, IFN-α production induced by CA-CpG was not only higher than that induced by K-type CpG ODNs but also higher than that induced by D-type CpG ODNs. Because IFN-α is a D-type CpG ODN-specific cytokine, these results suggest that CA-CpG-encapsulated K-type CpG ODNs gained characteristics of D-type CpG ODNs without losing their K-type CpG ODN characteristics. Many studies have shown that the difference between the cytokine production patterns induced by K-type and D-type CpG ODNs might be due to differences in their subcellular distribution in DCs (45, 46). It has been suggested that K-type CpG ODNs localize to late endosomes after cellular internalization and activate the IFN regulatory factor 5 pathway *via* TLR9, whereas D-type CpG ODNs localize to early endosomes and activate the IFN regulatory factor 7 pathway *via* TLR9 (47). In fact, a change in the localization of K-type CpG ODNs to early endosomes by the use of cationic liposomes has been shown to induce IFN-α production (47). In contrast, a new model was recently proposed in which adaptor protein 3-mediated TLR9 trafficking to lysosome-related organelles is essential for the induction of IFN-α production (48); this model suggests that the mechanisms of IFN-α production are more complicated than previously thought. Therefore, we cannot determine the precise relationship between the cellular localization of CA-CpG and its potential to induce IFN-α production. However, several studies have demonstrated that the particle size of CA-CpG affects its intracellular trafficking and localization. For example, small particles (<200 nm) are rapidly transported to late lysosomes, which have an acidic environment; whereas large particles (>500 nm) are localized in nearly neutral environments such as early endosomes and phagosomes (49, 50). These size-related differences may be the result of particle size-dependent differences in cellular internalization pathways (51). Therefore, we speculate that CA-CpG may localize in lysosome-related organelles, not in early endosomes, and may release CpG ODNs, which then induce IFN-α production. However, further study is required to clarify the exact cellular localization of CA-CpG.

In this study, we showed that, *in vitro*, CA-CpG was taken up by fewer DCs than were K-type CpG ODNs (Figures 3A,B). However, the number of CpG ODNs taken up by each DC was larger than the number of K-type CpG ODNs taken up by each DC, probably because CpG ODNs were condensed into a small space in the CA-CpG (Figures 3A,C). The greater uptake of CpG ODNs by cells treated with CA-CpG than by cells treated with K-type CpG ODNs might result in higher cytokine production in the former than in the latter.

With regard to the biodistribution of particles *in vivo*, several studies have suggested that small nanoparticles (<100 nm or <200 nm, depending on the study) can be trafficked to draining lymph nodes preferentially and can be taken up by DCs in lymph nodes, whereas larger particles (>100 nm or >200 nm, depending on the study) tend to be deposited at the injection site. These results suggest that nanoparticles <100 nm might be optimal for enhancing the efficacy of antigen and adjuvant delivery vehicles (52–54). We showed that the proportion of DCs that took up CpG ODNs in draining lymph nodes in CA-CpG-treated mice (8 µg CpG ODNs/mouse) was almost the same as the proportion in K-type CpG ODN-treated mice (8 µg CpG ODNs/mouse) (Figure 4A). However, cytokine production by lymph node cells was higher after administration of CA-CpG (8 µg CpG ODNs/mouse) than after administration of K-type CpG ODNs (8 µg CpG ODNs/mouse) (Figure 4B). These data suggest that, *in vivo* as well as *in vitro*, CA-CpG strongly induced cytokine production even though the number of CpG ODN-positive DCs was lower. It is not clear whether CpG ODN-positive individual DCs in the draining lymph nodes of CA-CpG-treated mice took up larger amounts of CpG ODNs than did individual DCs in the draining lymph nodes of K-type CpG ODN-treated mice. Further investigation of the biodistribution of CA-CpG *in vivo* is necessary.

The antigen (OVA and SV)-specific antibody responses (Figures 5A,B and 6) and CD8^+^ CTL responses (Figures 5C,D) induced by CA-CpG were superior to those induced by K-type CpG ODNs. The strength of the antigen-specific immune responses induced by CA-CpG was significantly higher than the strength of the responses induced by the same dose of K-type CpG ODNs and was almost the same as the strength of the responses induced by a dose of K-type CpG ODNs that was five times high. However, unlike K-type CpG ODNs (50 µg CpG ODNs/mouse), CA-CpG (10 µg CpG ODNs/mouse) did not induce splenomegaly, which suggests that CA-CpG may be a superior CpG ODN delivery vehicle in that it has better vaccine adjuvant effects than K-type CpG ODNs, without enhanced adverse effects (Figure 8). Many nanomaterials have the potential to enhance adaptive immunity, that is, they have adjuvant effects (55, 56). In this study, we found that the antibody responses in mice treated with SV plus CA nanoparticles without K-type CpG ODNs were slightly higher than the responses in mice treated with SV alone (Figures 6A,B). In contrast, the responses in mice treated with SV plus CA nanoparticles without K-type CpG ODNs were significantly lower than the responses in CA-CpG-treated mice (Figures 6A,B). These data suggest that the adjuvant activity of CA nanoparticles alone was low. In addition, we showed that CA-CpG performed better against influenza virus infection than did the same dose of K-type CpG ODNs or alum (Figure 6D). Following influenza infection, not only neutralizing antibodies but also CD8^+^ CTL responses play crucial roles in eliminating the virus and preventing viral persistence (57, 58). Furthermore, vaccines that induce CD8^+^ CTL responses are expected to show efficacy against influenza virus, because CD8^+^ CTL responses can provide cross-subtype or heterosubtypic protection (57, 58). Therefore, enhancement of CD8^+^ CTL responses by CA-CpG might lead to the development of a vaccine with strong protective effects against not only homologous but also heterologous influenza viruses. Taken together, these data suggest that CA-CpG can be used as a potent adjuvant for protein-based vaccines such as influenza SVs.

We showed that the enhancement of antibody responses and CD8^+^ CTL responses induced by CA-CpG tended to be negated in *Ifnar2-* and *Il-12 p40* double-deficient mice. We showed that CA-CpG induced the production of IFN-α and IL-12 in DCs more strongly than did K-type CpG ODNs *in vitro* (Figure 2). Previously, Kobiyama et al. showed that IFN-α and IL-12 are produced from plasmacytoid DCs and CD8α^+^ DCs, respectively, *in vitro* after stimulation with a complex consisting of CpG ODNs and β-glucan (19, 27). Furthermore, these investigators also showed that both IFN-α and IL-12 are indispensable for the induction of Th1-type antibody responses and CD8^+^ CTL responses induced by the CpG ODN complex (19, 27). These findings suggest that IFN-α produced by plasmacytoid DCs or IL-12 produced by CD8α^+^ DCs, or both, play important roles in the strong immune responses induced by CA-CpG.

In summary, we have shown that CpG ODNs encapsulated in CA nanoparticles as delivery vehicles induce enhanced cytokine production—especially IFN-α production—relative to that induced by CpG ODNs alone. Furthermore, we have shown CA-CpG is a potent adjuvant for protein-based subunit vaccines. We believe that the use of CA-CpG can improve the efficacy of influenza vaccines, such as vaccines for pandemic influenza and influenza vaccines for use among the elderly. In addition, CA-CpG may be superior to CpG ODNs as an adjuvant for vaccines against cancer, because CA-CpG can induce not only antigen-specific antibody responses but also CD8^+^ CTL responses. Thus, we believe that the novel pH-responsive CA nanoparticles reported herein have the potential to open up new avenues of research on vaccine adjuvant delivery vehicles. In addition, our results can be expected to lead to an improved understanding of the mechanisms of adjuvant effects and to the development novel particles or adjuvant delivery vehicles designed to improve vaccine efficacy.

## Ethics Statement

All animal experiments were performed in accordance with the institutional guidelines of Osaka University for the ethical treatment of animals. All experiments using human peripheral blood mononuclear cells were approved by the Institutional Review Board of the Research Institute for Microbial Diseases, Osaka University.

## Author Contributions

HT, TA, HY, and YY designed the experiments and interpreted the results. HT, KM, YYamamoto, and YK performed the experiments. HT, KM, YYamamoto, YK, and YY collected and analyzed the data. XW, EK, and KI provided technical support and conceptual advice. HT and YY wrote the manuscript. YY supervised the study.

## Conflict of Interest Statement

The following authors have conflict of interests to declare. TA, YYamamoto, and YYoshioka are employed by The Research Foundation for Microbial Diseases of Osaka University. HY and YYoshioka have filed a patent application related to the content of this manuscript. The other authors have no conflicts of interest to declare.

## Abbreviations

alum, aluminum salts; CA, carbonate apatite; CpG, cytosine-phosphate-guanine; CTL, cytotoxic T lymphocyte; DCs, dendritic cells; ELISA, enzyme-linked immunosorbent assays; FCS, fetal calf serum; HRP, Horseradish peroxidase; IFNs, interferons; Ifnar2, IFN-α/β receptor 2; IL, interleukin; ODNs, oligodeoxynucleotides; OVA, ovalbumin; PBMCs, peripheral blood mononuclear cells; SV, ether-treated hemagglutinin antigen-enriched virion-free split vaccine; TLR9, toll-like receptor 9.
